# Correction to Network module‐based drug repositioning for pulmonary arterial hypertension

**DOI:** 10.1002/psp4.13080

**Published:** 2023-11-14

Wang, R‐S, Loscalzo, J. Network module‐based drug repositioning for pulmonary arterial hypertension. *CPT Pharmacometrics Syst. Pharmacol*. 2021; 10: 994–1005. https://doi.org/10.1002/psp4.12670

In the subsection, “Consolidated human protein‐protein interactome,” the original article states, “This version of the consolidated human interactome has 16,470 proteins and 233,957 interactions, and displays a scale‐free topology.” Due to the inconsistency in gene nomenclature while compiling the human interactome, the number of proteins was mis‐reported. The actual amount should be 16,454 proteins. This minor error does not affect any of the results or conclusion in the paper.

We apologize for this error.

